# miRNA Signatures in Endometrial Cancer: Implications for Oncogenesis and Polymerase Epsilon (POLE) Mutation Status

**DOI:** 10.3390/ijms262110438

**Published:** 2025-10-27

**Authors:** Alexandros Lazaridis, Nikolas Dovrolis, Hector Katifelis, Despoina Myoteri, Iakovos Vlahos, Nikos F. Vlahos, Maria Gazouli

**Affiliations:** 1 2nd Department of Obstetrics and Gynecology, National and Kapodistrian University of Athens, Vasilissis Sofias 76, 11528 Athens, Greece; dr.alexlazaridis@gmail.com (A.L.); nfvlahos@gmail.com (N.F.V.); 2 Laboratory of Biology, Department of Basic Medical Sciences, Medical School, National and Kapodistrian University of Athens, Michalakopoulou 176, 11527 Athens, Greece; ndovroli@med.uoa.gr (N.D.); katifel@med.uoa.gr (H.K.); 3 Department of Pathology, Aretaieion Hospital, Medical School, National and Kapodistrian University of Athens, 76 Vas. Sofias Avenue, 11528 Athens, Greece; dmyoteri@med.uoa.gr (D.M.); iakvlachos@aretaieio.uoa.gr (I.V.)

**Keywords:** endometrial cancer, miRNAs, POLE mutation, biomarkers, gene regulation, prognosis

## Abstract

MicroRNAs (miRNAs) are key regulators of gene expression with critical roles in oncogenic signaling. Endometrial cancer (EC) has been redefined with the identification of POLE-ultramutated tumors which, despite their hypermutated phenotype, show more favorable prognosis. We profiled miRNA expression in tumor tissues from forty (40) EC patients and twenty (20) healthy controls using qPCR panels. POLE exonuclease domain mutations (P286R, V411L) were genotyped, and subgroup analyses were conducted between POLE-mutated (n = 7) and POLE-wild-type (n = 33) tumors. Bioinformatic analyses included validated miRNA–mRNA interactions, target enrichment, and Gene Ontology (GO) pathway mapping. Comparison of EC versus healthy endometrium revealed 50 significantly dysregulated (∣log_2_ (FoldReg)∣ > 1 and BH FDR < 0.05) miRNAs, including up-regulation of the oncogenic hsa-miR-181a-5p, hsa-miR-23a-3p, hsa-miR-200c-3p, and down-regulation of tumor-suppressive let-7 family members. Target enrichment implicated canonical oncogenic regulators such as *MYC*, *TP53*, and *VEGFA*. POLE-mutated tumor analysis demonstrated a miRNA signature, with 19 miRNAs significantly down-regulated, including let-7f-5p and hsa-miR-200b-3p. Findings for the EC versus healthy endometrium comparison were validated against TCGA-UCEC sequencing data which confirmed concordant dysregulation of key miRNAs across platforms. Our findings reveal that EC is characterized by widespread miRNA deregulation, with a unique global down-regulation signature in POLE-mutated tumors. These results highlight the potential of miRNAs as complementary biomarkers for classification and potential targets in EC.

## 1. Introduction

MicroRNAs (miRNAs) are short non-coding RNA molecules, usually between 19 and 25 nucleotides in length, that regulate gene expression post-transcriptionally. By guiding the silencing of messenger RNAs, they exert broad influence over cell cycle progression, apoptosis, differentiation, and stress responses. Given that such pathways are frequently deregulated in cancer, it is unsurprising that altered miRNA expression has been documented across virtually every tumor type described to date. Some miRNAs promote oncogenic signaling (so-called oncomiRs), while others act as tumor suppressors, and their net effect depends on the balance of targets within a given cellular context [1,2,3].

Endometrial cancer (EC) represents the most common gynecological malignancy in industrialized developed countries, exhibiting a strong association with lifestyle; therefore, its incidence continues to rise in parallel with increasing rates of obesity and metabolic syndrome [4]. Histopathology alone has long been recognized as an incomplete predictor of prognosis, and over the past decade, large-scale genomic efforts, most notably The Cancer Genome Atlas (TCGA), have reshaped our understanding of EC biology. The TCGA analysis defined four molecularly distinct subgroups: DNA Polymerase Epsilon (POLE)-ultramutated, microsatellite instability-high (MSI-H), copy-number low (endometrioid), and copy-number high (serous-like) [5]. These molecular classes are now embedded into risk-stratification algorithms, but considerable heterogeneity remains within each group, pointing to the need for necessitating the identification and adoption of widespread complementary biomarkers.

A growing body of work implicates miRNAs in nearly every stage of EC pathogenesis. It has become common knowledge that the aberrant expression of the miR-200 family has been associated with epithelial–mesenchymal transition and invasion [6,7], while miR-34a which regulates p53-dependent checkpoints, is frequently downregulated [8]; and miR-21, arguably the archetypal oncomiR, is consistently upregulated, targeting PTEN and other tumor suppressors [9]. Beyond tissue profiling, stable miRNAs in endometrial fluid have been proposed as minimally invasive biomarkers for early diagnosis and disease monitoring [10]. Nevertheless, discrepancies between studies remain, in part due to differences in experimental design, platforms used for miRNA detection, and clinical heterogeneity of patient cohorts.

Within the molecular taxonomy of EC, tumors harboring the pathogenic variants in the exonuclease domain of DNA polymerase epsilon (*POLE*) are particularly intriguing. Despite accumulating hundreds of mutations per megabase due to defective proofreading, these POLE-ultramutated carcinomas paradoxically behave in an indolent manner and are associated with excellent long-term survival [11,12,13]. The most common hotspot mutations—*P286R* and V411L—lie within the exonuclease domain, disrupting the polymerase’s fidelity [12]. Several studies have described a distinctive mutational landscape in *POLE*-mutated EC, including alterations in *TP53*, *PIK3CA*, and *CTNNB1*, and a highly immunogenic tumor microenvironment which may help explain their favorable prognosis [5,14,15]. Biologically, *POLE* mutations lead to defective proofreading, with subsequent accumulation of a multitude mutations thus representing a hypermutated phenotype. Intriguingly, despite their genomic instability, these tumors are associated with excellent favorable progression-free and overall survival, a paradox thought to be mediated by enhanced immunogenicity and increased tumor-infiltrating lymphocytes [16,17].

However, while genomic correlations of the *POLE* status are well-characterized, little is known about how miRNA expression integrates with this molecular subtype. Given the regulatory capacity of miRNAs across oncogenic and immune-related pathways, their expression may offer critical insights into the unique biology of *POLE*-mutated EC. In light of these observations, the present study aimed to comprehensively characterize miRNA expression profiles in endometrial cancer, with a dual focus on their role in oncogenesis and their association with *POLE* mutation status. By integrating miRNA profiling with the molecular classification, we seek to clarify the regulatory networks underlying EC progression and to evaluate the potential of miRNAs as biomarkers for risk stratification and therapeutic decision-making in *POLE*-mutated disease.

## 2. Results

### 2.1. Differential Expression of miRNAs Between Cancer and Healthy Groups

**Figure 1:**
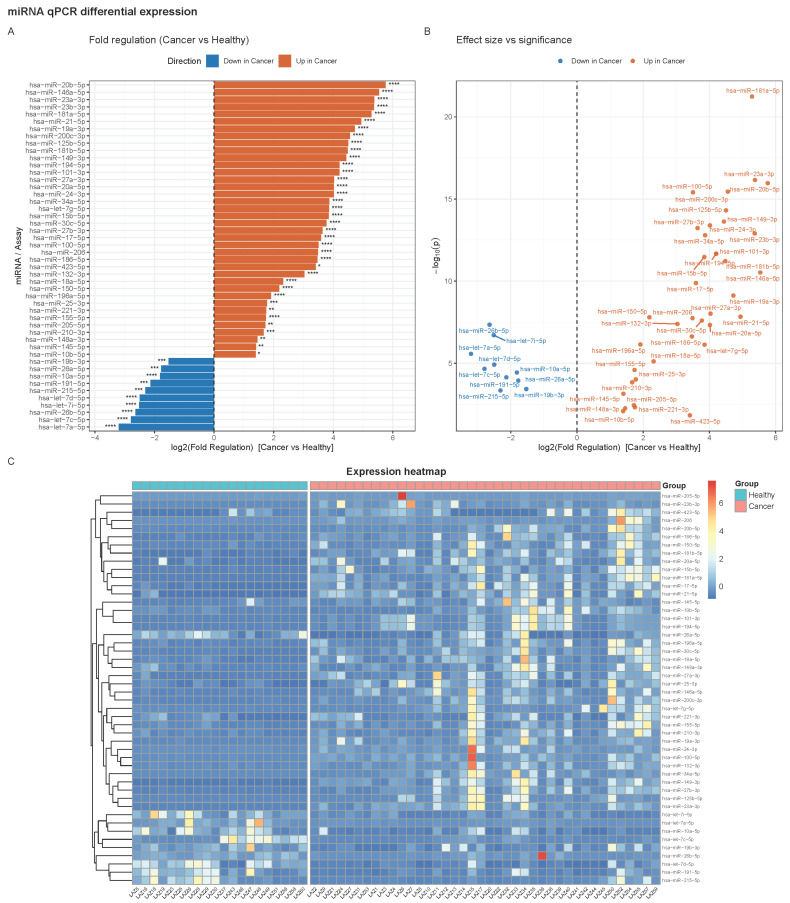
miRNA qPCR differential expression across healthy and cancer cohorts. Group sizes: healthy n = 20, cancer n = 40. (**A**) Fold-regulation barplot (cancer vs. healthy). Bars show log_2_ (fold regulation) for miRNAs passing the display threshold |log_2_FC| > 1 (i.e., ≥2-fold change in either direction). Bars are colored by direction (Up in cancer vs. Down in cancer). The dashed vertical line marks no change (log_2_FC = 0). Asterisks denote significance based on BH-FDR unless otherwise noted: * q < 0.05, ** q < 0.01, *** q < 0.001, **** q < 1 × 10^−4^. (**B**) Volcano plot (filtered set). Each point corresponds to a plotted miRNA (same |log_2_FC| > 1 subset as in (**A**); the *x*-axis is log_2_ (fold regulation) and the *y*-axis is–log_10_ (*p*-value) from Welch’s *t*-test on ΔCt. Points are colored by direction (up/down in cancer). The vertical dashed line marks log_2_FC = 0. Labels are shown for miRNAs meeting FDR < 0.05. (**C**) Heatmap of normalized expression across all samples. Values are z-scores of 2^−ΔCt^ computed row-wise (per miRNA) to emphasize relative variation across samples. Columns are ordered by group with a visual gap; rows are hierarchically clustered. Only non-housekeeping miRNAs that passed |log_2_FC| > 1 are displayed.

After ΔCt normalization and group comparison, 50 miRNAs showed significant differential expression between cancer (n = 40) and healthy (n = 20) specimens at |log_2_ (FoldReg)| > 1 and FDR < 0.05. Of these, 38 miRNAs were up-regulated in endometrial cancer, while 10 were down-regulated. Among the most significantly up-regulated miRNAs were hsa-miR-181a-5p (log_2_FC = +5.28, FDR = 4.9 × 10^−20^), hsa-miR-23a-3p (+5.37, FDR = 3.0 × 10^−15^), hsa-miR-20b-5p (+5.76, FDR = 3.1 × 10^−15^), hsa-miR-100-5p (+3.50, FDR = 6.8 × 10^−15^), hsa-miR-200c-3p (+4.56, FDR = 6.8 × 10^−15^), and hsa-miR-125b-5p (+4.50, FDR = 7.1 × 10^−14^). Other strongly up-regulated candidates included miR-149-3p, miR-24-3p, miR-27b-3p, and miR-23b-3p, all with log_2_ fold changes >3.5 and FDR < 1 × 10^−12^. By contrast, the down-regulated set was dominated by members of the let-7 family. These included hsa-let-7a-5p (log_2_FC = −3.20, FDR = 7.4 × 10^−6^), hsa-let-7c-5p (−2.79, FDR = 5.3 × 10^−5^), hsa-let-7d-5p (−2.50, FDR = 3.1 × 10^−5^), and hsa-let-7i-5p (−2.51, FDR = 6.4 × 10^−7^). Additional significantly down-regulated miRNAs included miR-26b-5p (−2.64, FDR = 1.6 × 10^−7^), miR-26a-5p (−1.77, FDR = 2.4 × 10^−4^), miR-19b-3p (−1.53, FDR = 7.6 × 10^−4^), and miR-191-5p (−2.13, FDR = 1.6 × 10^−4^) (Figure 1A).

Given the observed variability of ΔCt values (SD ≈ 1.8), the Cancer vs. Healthy comparison (n = 40 vs. 20) had sufficient sensitivity to detect moderate expression changes. At 80% power, the minimal detectable effect size (MDES) was 2.23 log_2_ units (~4.7-fold) under a Bonferroni correction and 1.47 log_2_ units (~2.8-fold) under the BH-effective α; at 90% power, these thresholds increased to 2.46 (~5.5-fold) and 1.69 (~3.2-fold), respectively. These values indicate that the Cancer vs. Healthy comparison was adequately powered to detect biologically relevant changes typical of qPCR miRNA studies (Supplementary Materials).

Volcano plots (Figure 1B) revealed a clear bifurcation of the effect sizes with both extreme up- and down-regulated miRNAs, while the supervised heat map demonstrated consistent clustering by disease status, with cancer samples enriched for oncomiRs and healthy samples showing higher expression of tumor-suppressive let-7 family members (Figure 1C).

To evaluate whether clinical variables influenced miRNA expression, we performed a multivariate linear model in R v.4.4.0 (lm) including age, body mass index (BMI), and tumor grade as covariates for the top 10 dysregulated miRNAs. None of these factors significantly affected the association between disease status and miRNA expression (all adjusted *p* > 0.1), indicating that the observed differences were independent of these potential confounders.

### 2.2. miRNA–Gene Target Convergence

**Figure 2:**
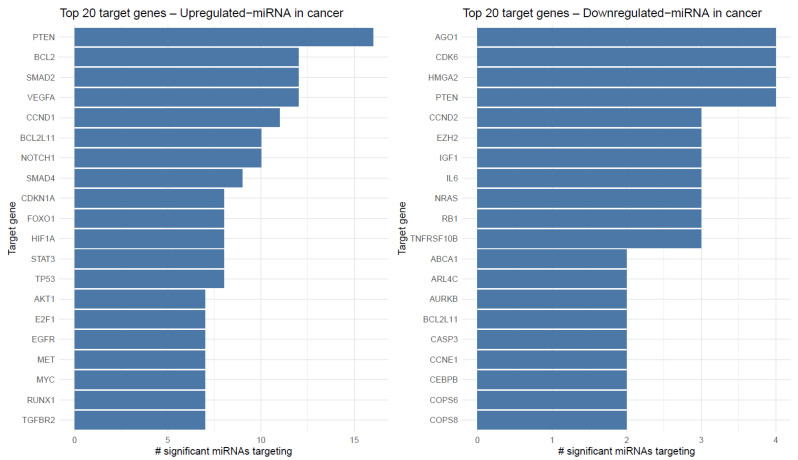
Top validated target genes of differentially expressed miRNAs. For each direction, bars rank target genes by the number of distinct significant miRNAs that have validated interactions with that gene (unique miRNA-gene pairs). X-Axis labels indicate the count of significant miRNAs per gene.

Target enrichment analysis highlighted that up-regulated miRNAs in cancer converged on canonical oncogenic drivers. The most frequently targeted genes included *TGFBR2*, *RUNX1*, *MYC*, *MET*, *EGFR*, *E2F1*, *AKT1*, *TP53*, *STAT3*, *HIF1A*, and *VEGFA*, each regulated by multiple significant miRNAs. Down-regulated miRNAs, in contrast, mapped to targets such as *RB1*, *NRAS*, *IL6*, *CDK6*, *CCNE1*, *CASP3*, and *EZH2*, suggesting potential derepression of cell cycle and survival pathways (Figure 2).

### 2.3. Functional Enrichment of Target Gene Sets

**Figure 3:**
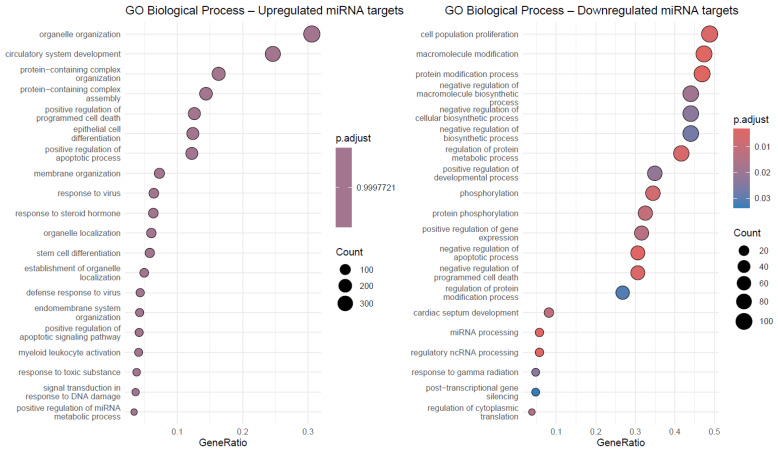
GO Biological Process enrichment of validated miRNA targets. Dotplots display the top 20 GO:BP categories for targets of up-regulated and down-regulated miRNAs, respectively. The background universe is the union of all validated targets from the significant miRNAs. Dot size reflects the number of target genes mapped to each term (Count), and color encodes the adjusted *p*-value returned by enriched GO.

Gene Ontology (GO) Biological Process enrichment revealed that up-regulated miRNAs were linked to pathways including signal transduction in response to DNA damage, myeloid leukocyte activation, apoptotic signaling, viral defense responses, and epithelial cell differentiation. On the opposite end, down-regulated miRNAs were associated with processes such as post-transcriptional gene silencing, miRNA processing, regulation of protein phosphorylation, and negative regulation of apoptosis, indicating broad derepression of translational and metabolic programs (Figure 3).

### 2.4. Differential Expression of miRNAs Between POLE^+^ and POLE^−^ Tumors

**Figure 4:**
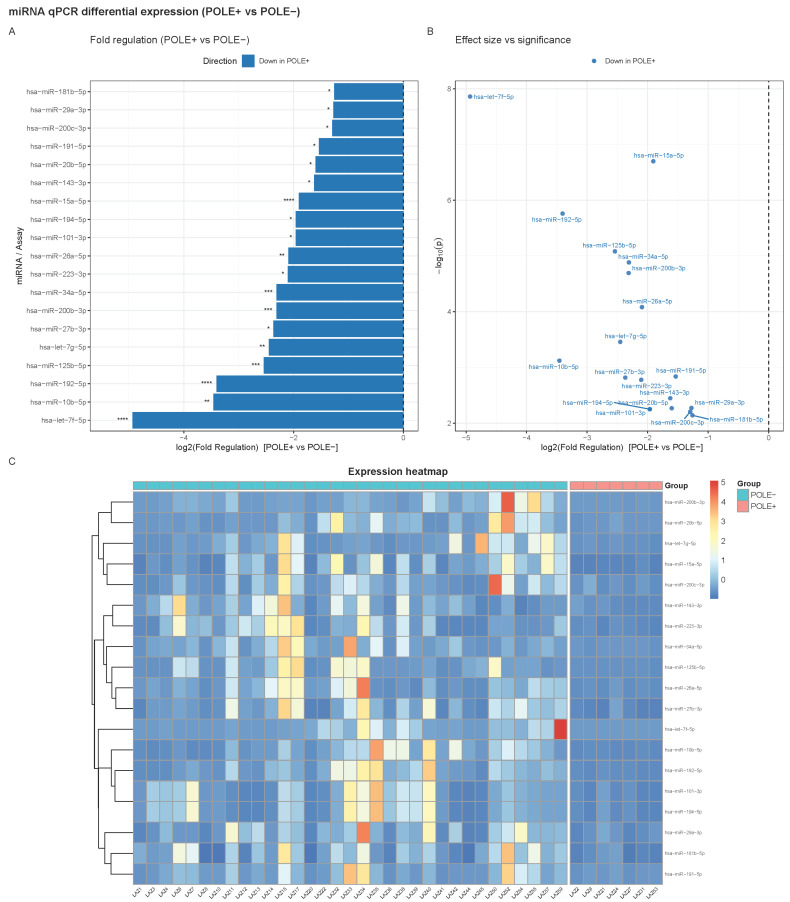
Differential miRNA expression in POLE-mutated (POLE+) versus POLE-wild-type tumors (POLE^−^). (**A**) Bar plot of log_2_ fold regulation values showing significant miRNAs down-regulated in POLE-mutated tumors compared with POLE^−^ cases. Asterisks denote significance based on BH-FDR unless otherwise noted: * q < 0.05, ** q < 0.01, *** q < 0.001, **** q < 1 × 10^−4^. (**B**) Volcano plot illustrating effect size versus significance, highlighting the absence of up-regulated miRNAs in the POLE+ group. (**C**) Heat map of normalized expression (2^−ΔCt^ z-scores) across all samples, grouped by POLE status, showing consistent reduction in specific miRNAs in POLE-mutated tumors.

In our cohort, most POLE-mutated tumors (n = 7) carried at least one heterozygous point mutation, and comparison of POLE-mutated tumors (n = 7) against POLE-wild-type tumors (n = 33) identified 19 significantly dysregulated miRNAs at |log_2_ (FoldReg)| > 1 and FDR < 0.05. Strikingly, all significant changes reflected down-regulation in POLE-mutant cases, with no miRNAs showing significant up-regulation. The strongest reductions were observed for hsa-let-7f-5p (log_2_FC = −4.93, FDR = 1.0 × 10^−6^), hsa-miR-192-5p (−3.41, FDR = 5.0 × 10^−5^), hsa-miR-10b-5p (−3.46, FDR = 7.3 × 10^−3^), and hsa-miR-125b-5p (−2.54, FDR = 1.8 × 10^−4^). Additional significantly down-regulated miRNAs included miR-34a-5p (−2.31, FDR = 2.3 × 10^−4^), miR-200b-3p (−2.31, FDR = 2.9 × 10^−4^), miR-26a-5p (−2.09, FDR = 1.0 × 10^−3^), miR-15a-5p (−1.91, FDR = 9.0 × 10^−6^), let-7g-5p (−2.45, FDR = 3.8 × 10^−3^), and miR-191-5p (−1.54, FDR = 1.2 × 10^−2^). The bar plot, volcano plot, and heatmap panels consistently reflected this global down-regulation of miRNAs in the POLE^+^ group, with no significant evidence for compensatory up-regulation (Figure 4). This might be largely attributed to the statistical power of the sample size where the POLE^+^ vs. POLE^−^ comparison (n = 7 vs. 33) was underpowered for smaller effects: the 80% MDES was 3.44 (~10.9-fold) under Bonferroni and 2.36 (~5.1-fold) under BH-effective α, reaching 3.80 (~13.9-fold) and 2.70 (~6.5-fold) at 90% power.

**Figure 5:**
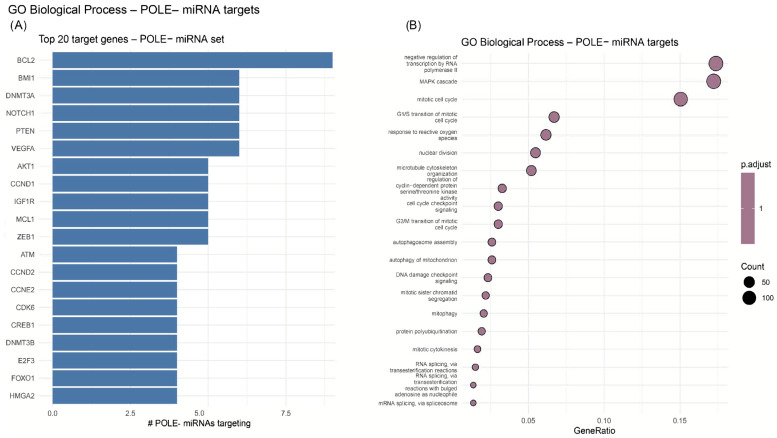
Predicted gene targets and pathways of miRNAs down-regulated in POLE-mutated tumors. (**A**) Top 20 genes most frequently targeted by the down-regulated miRNAs, (**B**) enriched Gene Ontology Biological Process (GO:BP) terms.

Validated target enrichment revealed distinct convergence among POLE+ downregulated miRNAs. For miRNAs down in POLE+, predicted target convergence implicated central oncogenic regulators, including *HMGA2*, *FOXO1*, *E2F3*, *DNMT3B*, *CREB1*, *CDK6*, *CCNE2*, *CCND2*, *ATM*, *ZEB1*, *MCL1*, *IGF1R*, *CCND1*, *AKT1*, *VEGFA*, *PTEN*, *NOTCH1*, *DNMT3A*, *BMI1*, and *BCL2* (Figure 2). No validated targets were detected for up-regulated miRNAs, consistent with the absence of significant up-regulation in POLE+ (Figure 5A). GO Biological Process enrichment analysis for the POLE+ miRNA set indicated strong enrichment in pathways governing RNA splicing (via spliceosome and transesterification reactions), mitotic cytokinesis, sister chromatid segregation, DNA damage checkpoint signaling, MAPK cascade, and cell cycle transitions (G1/S, G2/M). Additional processes included mitophagy, autophagy, and response to reactive oxygen species, suggesting broad suppression of miRNAs involved in genome stability and mitochondrial quality control (Figure 5B).

### 2.5. Validation Against TCGA miRNA-Seq

**Figure 6:**
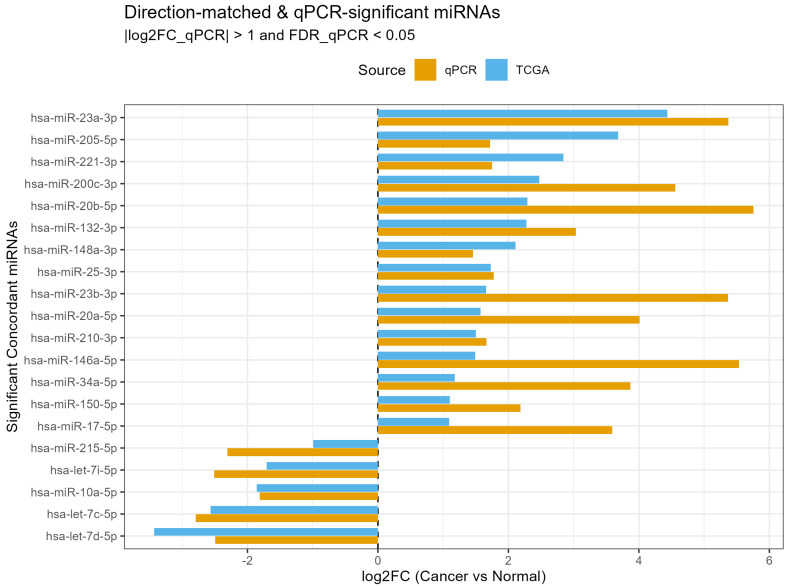
Comparison of qPCR Cancer vs. Healthy and TCGA-UCEC miRNA expression. The barplot displays only miRNAs that were concordant and statistically significant in qPCR (|log_2_FC| > 1, FDR < 0.05), with side-by-side log_2_FC values from qPCR (orange) and TCGA (blue).

For externally validating the qPCR findings, we compared the Cancer vs. Healthy differential expression qPCR profile to TCGA-UCEC miRNA sequencing data. Across datasets, 20 miRNAs showed statistically significant and directionally concordant dysregulation. Key examples include the strong up-regulation of miR-23a-3p, miR-205-5p, miR-221-3p, miR-200c-3p, miR-20b-5p, and miR-210-3p, as well as the consistent down-regulation of let-7a-5p, let-7c-5p, let-7d-5p, let-7i-5p, and miR-10a-5p. While effect size magnitudes varied somewhat between qPCR and TCGA (likely due to platform-specific dynamic ranges), the directional consistency across methods strengthens the robustness of the observed miRNA signature. More than half of the overlapping miRNAs (55.4%) showed concordant direction of regulation between qPCR validation and TCGA-UCEC sequencing data which is within the range expected for cross-platform comparisons and supports the robustness of our experimental findings. The final visualization focused on miRNAs that were both concordant in direction and statistically significant in the qPCR dataset (|log_2_FC| > 1 and FDR < 0.05). These miRNAs were plotted in a grouped barplot, showing side-by-side log_2_FC estimates from TCGA-UCEC and qPCR, to highlight robust agreement between these public sequencing data and experimental validation (Figure 6).

## 3. Discussion

In this study, we identified a distinct miRNA expression signature that differentiates EC from healthy tissue. After ΔCt normalization and rigorous statistical adjustment, 50 miRNAs were found to be significantly dysregulated, with most showing increased expression in EC specimens. Several well-characterized oncogenic miRNAs (oncomiRs), including hsa-miR-181a-5p, hsa-miR-23a-3p, hsa-miR-20b-5p, hsa-miR-100-5p, hsa-miR-200c-3p, and hsa-miR-125b-5p, exhibited strong up-regulation, consistent with their established roles in promoting tumor proliferation, invasion, and epithelial–mesenchymal transition (EMT) in other malignancies. Our findings are generally in concordance with previous studies, supporting the implication of these miRNAs in endometrial tumor biology and epithelial–mesenchymal plasticity [18,19,20,21]. The literature on hsa-miR-23a-3p remains controversial, with some reports describing tumor-suppressive effects through reduced expression and EMT restraint, while others (including serum-based studies in gynecologic malignancies) report up-regulation. These discrepancies may reflect tumor histology, sample type, or cohort differences. Our findings support its up-regulation in tumor tissue [22].

In contrast, the most markedly down-regulated group in our dataset comprised members of the hsa-let-7 family. This finding is consistent with prior studies in EC, where diminished let-7 activity has been linked to the release of oncogenic pathways such as RAS, MYC, and HMGA, thereby promoting uncontrolled proliferation. Both early and recent reports, along with functional experiments in EC cells, consistently highlight the tumor-suppressive role of hsa-let-7. Our findings therefore reinforce the down-regulation of this family as a recurrent hallmark of endometrial carcinogenesis [23]. Beyond the hsa-let-7 family, we observed significant down-regulation of miR-26b-5p, miR-26a-5p, miR-19b-3p, and miR-191-5p, pointing to broader attenuation of miRNA-mediated tumor suppression in EC. Loss of the miR-26 family has been described in EC and other cancers, where it relieves cell cycle and epigenetic checkpoints through targets such as CCND2 and EZH2 [24]. Similarly, miR-19b-3p has been reported to function as a tumor-suppressive miRNA in EC by regulating MYCN [25]. Finally, miR-191-5p was notably reduced in EC, with lower levels in higher-grade tumors, supporting its proposed tumor-suppressive role [26].

Target enrichment analysis showed that dysregulated miRNAs converge on major oncogenic hubs. Up-regulated miRNAs collectively targeted genes such as *TGFBR2*, *RUNX1*, *MYC*, *MET*, *EGFR*, *E2F1*, *AKT1*, *TP53*, *STAT3*, *HIF1A*, and *VEGFA* which are central to PI3K/AKT–mTOR, MAPK/EGFR, and hypoxia-driven angiogenesis pathways—well-established contributors to EC progression. [27] In contrast, down-regulated miRNAs mapped to *RB1*, *NRAS*, *IL6*, *CDK6*, *CCNE1*, *CASP3*, and *EZH2*, consistent with derepression of cell cycle progression, survival, and epigenetic remodeling, mechanisms supported in EC models, including *EZH2*-driven miRNA silencing and sensitivity to EZH2 inhibition [28]. Gene Ontology analysis reflected this dichotomy: up-regulated miRNA targets were enriched in DNA damage responses, leukocyte activation, apoptosis, antiviral defense, and epithelial differentiation, while down-regulated targets mapped to post-transcriptional silencing, miRNA processing, regulation of protein phosphorylation, and inhibition of apoptosis. Together, these findings suggest a coordinated reprogramming in which proliferative and inflammatory pathways are amplified, while multiple miRNA-dependent checkpoints are simultaneously suppressed [29].

POLE mutations, particularly within the exonuclease domain, are known to confer an ultramutated phenotype in endometrial cancer, characterized by exceptionally high tumor mutational burden and a favorable prognosis. These tumors often display increased neoantigen load and robust immune infiltration which distinguish them from other molecular subtypes of EC [5]. In our series, POLE-positive tumors showed uniform down-regulation of 19 miRNAs (none up-regulated), including let-7f-5p, hsa-miR-192-5p, hsa-miR-10b-5p, hsa-miR-125b-5p, hsa-miR-34a-5p, hsa-miR-200b-3p, hsa-miR-26a-5p, hsa-miR-15a-5p, let-7g-5p, and hsa-miR-191-5p. The functional footprint of these losses is coherent with known tumor-suppressive circuits in EC such as diminished let-7 which permits HMGA2/RAS–MYC activity, reduced hsa-miR-34a which weakens p53-dependent control and associates with invasive traits, and loss of hsa-miR-200b which relaxes repression of ZEB1/2 and EMT [30]. Target convergence in POLE-positive tumors included E2F3, DNMT3B, CDK6/CCNE2, ATM, AKT1/VEGFA, PTEN, and BCL2, pointing to derepression of cell cycle progression, DNA-damage response, angiogenesis, and apoptotic escape—hallmarks of ultramutated EC [5] GO analysis showed enrichment of pathways related to RNA processing, mitotic cytokinesis, DNA damage signaling, MAPK cascades, autophagy, and oxidative stress responses, suggesting that global miRNA loss in POLE-positive tumors may represent an adaptation to replication stress and genome instability [5]. This POLE-specific global suppression differs from the broader EC pattern observed in the cancer-versus-healthy comparison, where prominent oncomiR up-regulation (miR-200 family, miR-181a, miR-20b) occurred alongside let-7 loss. The miR-200 family is strongly linked to EMT regulation via ZEB1/2 repression, while miR-181a and miR-20b are associated with proliferative and diagnostic roles in EC [31]. In parallel, loss of let-7 unleashes HMGA2 and RAS/MYC signaling, a traditional tumor-suppressive axis described across epithelial tumors and reported in gynecologic contexts [30]. Against this backdrop, the POLE^+^ subtype appears to achieve pathway activation primarily through system-wide release of miRNA-mediated brakes that is consistent with the replication stress, ultrahigh mutational burden, and heightened immune activity characteristic of POLE-mutant EC, rather than by further amplifying oncomiRs. [32] This contrast supports our finding that POLE^+^ tumors are not distinguished by selective oncomiR up-regulation but rather by a coordinated suppression of multiple tumor-restraining miRNAs, leading to derepression of cell cycle, DNA-damage, and survival networks [19]. A limitation of our study is the relatively small number of POLE-mutant tumors in our cohort (n = 7 vs. 33 POLE-wild-type) which may constrain statistical power and increase the risk of type I/II error in differential expression analyses. This reflects the known rarity of POLE exonuclease-domain mutations in endometrial cancer, typically reported in ~7–12% of cases in large sequencing cohorts such as TCGA [33]. Despite the small sample size, the consistent and uniform down-regulation observed across all POLE-mutant tumors, along with strong convergence on canonical cell cycle and DNA-damage regulators, supports the biological validity of this signal. Nonetheless, these findings should be interpreted with caution and ideally validated in larger, independent cohorts.

In addition, several miRNAs, such as hsa-miR-20b-5p, exhibited divergent expression patterns when comparing cancer versus healthy tissue and POLE^+^ versus POLE^−^ tumors. Specifically, miR-20b-5p was up-regulated in endometrial cancer relative to normal endometrium but relatively down-regulated within the POLE^+^ subset. This apparent inversion likely reflects context-dependent regulation: while miR-20b-5p promotes proliferative and epithelial–mesenchymal transition (EMT) pathways during tumorigenesis, its suppression in POLE-mutated tumors may correspond to the global miRNA down-regulation phenotype that characterizes ultramutated cancers. Such context-dependent shifts highlight that miRNA expression reflects not only malignant transformation but also the molecular background of the tumor subtype. Therefore, POLE mutation testing remains biologically and clinically relevant, as miRNA profiling provides complementary, not substitutive, information that refines molecular subclassification beyond histopathology alone. Together, these findings emphasize the complex regulatory interactions that distinguish POLE^+^ tumors from other EC subtypes and warrant further functional exploration.

Beyond confirming the miRNA dysregulation patterns reported in TCGA, our most distinctive finding lies in the POLE-mutated subset, which displayed a coherent and global down-regulation of miRNAs. This widespread suppression was consistent across nearly all significant features, including members of the let-7, miR-34, miR-200, and miR-26 families—key regulators of cell cycle control, DNA damage response, and apoptosis. Such coordinated down-regulation suggests a systemic attenuation of miRNA-mediated gene silencing, potentially reflecting adaptation to replication stress, increased mutational load, or altered chromatin dynamics characteristic of ultramutated tumors. Notably, this signature was evident despite the small sample size (n = 7 POLE^+^ tumors), emphasizing its robustness and biological significance. These findings suggest that the POLE-ultramutated subtype is not simply a quantitative extension of canonical EC deregulation but represents a qualitatively distinct regulatory state. By highlighting this unique global down-regulation profile, our results contribute novel insight into the post-transcriptional landscape of POLE-driven endometrial tumorigenesis.

The observed global suppression of miRNA expression in POLE-mutated tumors may parallel transcriptional consequences described in other malignancies harboring POLE exonuclease-domain mutations. Recent studies in colorectal cancer have demonstrated that POLE mutations can reshape the mRNA expression landscape, supporting the concept of mini-driver genes that exert subtle yet functionally relevant transcriptional effects across multiple pathways [32]. Within this framework, our results suggest that POLE-driven endometrial cancers may undergo a comparable form of transcriptional reprogramming, extending from mRNA to the miRNA regulatory layer. The consistent down-regulation of key tumor-suppressive miRNAs (let-7, miR-34, miR-200 families) implies a coordinated relaxation of post-transcriptional control that may act in concert with hypermutation-induced transcriptional noise. These findings highlight a potential mechanistic continuum between DNA polymerase dysfunction and downstream gene-regulatory alterations, positioning POLE as a driver of genome-wide regulatory imbalance in endometrial cancer.

Finally, we confirmed our Cancer vs. Healthy results through external validation with TCGA-UCEC data. Twenty miRNAs were significantly and directionally concordant between qPCR and sequencing datasets, including up-regulation of hsa-miR-23a-3p, hsa-miR-205-5p, hsa-miR-221-3p, hsa-miR-200c-3p, hsa-miR-20b-5p, and hsa-miR-210-3p, and down-regulation of let-7 family members and hsa-miR-10a-5p. These findings mirror established roles of these miRNAs in EC biology. Although effect sizes varied between platforms, the >55% concordance rate is in line with cross-platform expectations and highlights the robustness of our signature [34]. By focusing on the subset of miRNAs both statistically significant and concordant across qPCR and TCGA, we highlight a reliable panel of dysregulated miRNAs in EC, strengthening confidence in their biological and translational relevance [35].

In summary, our study defines a reproducible miRNA expression signature that distinguishes EC from normal tissue, integrates and aligns with known oncogenic pathways, and highlights distinct patterns in POLE-mutant tumors. These findings reinforce the biological importance of miRNA dysregulation in EC and nominate a core set of miRNAs with potential utility as biomarkers and therapeutic targets [27,28,33,34].

## 4. Materials and Methods

### 4.1. Patients and Tissue Samples

This prospective study was conducted at Aretaieion University Hospital, 2nd department of Obstetrics and Gynaecology of the National and Kapodistrian University of Athens, Greece; from February 2024 till April 2025. Sixty (60) participants were included, of which forty (40) consecutive cases of endometrial cancer served as the study group. Inclusion criteria were all adult women over the age of 18 years old with endometrial cancer confirmed on histology, following pipelle endometrial sampling or hysteroscopy with guided targeted endometrial biopsy or the traditional method of dilation and curettage. Moreover, we also matched and included twenty (20) cases with normal endometrium that underwent hysterectomy for pelvic organ prolapse (mean age 65.25 years old, youngest 45 and oldest 78 years old) that served as the control group. The exclusion criteria for the control group were any current known cervical, endometrial, or myometrial pathology, such as endometriosis, adenomyosis, significant uterine fibroid volume, or previous cervical intraepithelial neoplasia (CIN) or endometrial hyperplasia that subsequently regressed. Thus, we recruited women with pre-operative sonographic evidence of normal sized uterus, and atrophic endometrium with a cut-off endometrial thickness below 4 mm was considered for all menopausal women [35]. Each cancer patient had a confirmatory pre-operative histological diagnosis as well as a pelvic magnetic resonance imaging (MRI) and chest–abdomen computed tomography (CT) scan to aid in the surgical staging according to the revised FIGO guidelines [36]. All patients were discussed at our departmental gynae-oncology multidisciplinary team (MDT) meetings, and written informed consent was obtained from all participants (after approval by the departmental research & ethics committee-approval no. 553/2 February 2024). We collected uterine samples from all sixty (60) participants. The demographic and clinicopathological data of the participants are presented in Table 1.

Immediately following the uterine extraction from the body, the whole specimen was transported at the histopathology laboratory, whereby the dedicated team extracted endometrial samples which were stored at RNA Later (Qiagen, Hilden, Germany) for subsequent, more definitive testing and/or future use according to the manufacturer’s instructions. Then, the remaining sample (cervix and uterus) were immersed and fixated into formalyn for the conclusive histopathological and immunohistochemical analysis. Additionally, pre-operative peripheral blood was collected from all the participants. All patients were treatment-naïve at the time of sample collection. No patient had received chemotherapy, radiotherapy, or hormonal therapy prior to surgery. All tissue specimens were therefore collected before any therapeutic intervention to avoid treatment-related alterations in miRNA expression. The demographic and clinicopathological characteristics of the study population are summarized in Table 2. Survival or treatment-response data were not available at the time of analysis due to the short follow-up period, and this is acknowledged as a limitation of the study.

### 4.2. POLE Genotyping

Genomic DNA was extracted from blood samples using the NucleoSpin Blood Kit (Macherey-Nagel, Düren, Germany) according to the manufacturer’s instructions. The quality and concentration of purified DNA was estimated using the NanoDrop 8000 Spectrophotometer (Thermo Fisher Scientific Inc., Waltham, MA, USA). We genotyped our samples for *POLE* exonuclease domain mutations, focusing on the two most common alterations associated with ultramutated endometrial carcinomas: P286R (c.857 C > G, exon9), and V411L (c.1231G > T/C, exon13), known hotspot mutations in the exonuclease domain of the *POLE* gene [37]. Genotyping of the hotspot mutations P286R (c.857 C > G, exon9), and V411L (c.1231G > T/C, exon13), was performed using allele-specific PCR. The sequences of the primers, along with the product length and annealing temperatures are listed in Table 2.

### 4.3. MiRNA Expression and Bioinformatics Analysis

MiRNA isolation was carried out from tissue samples with the NucleoSpin miRNA kit (Machnery-Nagel, Düren, Germany), and miRNAs were profiled with a 96-plex miRCURY LNA miRNA PCR Panel (Qiagen, Cat. no. YAHS-102Y) following the manufacturer’s instructions, including reverse transcription with the miRCURY LNA RT Kit (Qiagen) and amplification with the miRCURY LNA SYBR Green Master Mix (Qiagen). The panel includes reference (housekeeping) miRNAs and proprietary assay controls to monitor reverse transcription and PCR efficiency. A Ct lower limit of detection was defined at 35 cycles by the acquisition software, and, in this dataset, per-sample missingness was 0% across all 60 runs. Unsupervised quality control provided by the GeneGlobe portal (scatter/volcano overviews, PCA, and a clustergram) was used to corroborate group separation and to inspect outliers. On these quality-controlled sample data, we performed two distinct groupings and similar downstream analyses. The first one focused on cancer samples vs. healthy controls, while the other delved into the miRNA expression differences between the original tumor samples with the supermutated POLE (n = 7) and those without (used as controls; n = 33). The downstream analyses are as follows:

For within-sample normalization, ΔCt was computed for each assay as Ct minus the arithmetic mean Ct of three housekeeping references (SNORD38B, U6 snRNA, SNORD49A) measured in the same sample. Relative expression was defined as 2^−ΔCt^. To summarize group effects, ΔΔCt was calculated per miRNA as the difference between the group means of ΔCt (e.g., Cancer minus Healthy), from which fold regulation was derived as 2^−ΔΔCt^ and reported on the log_2_ scale. By this convention, positive log_2_(FoldReg) indicates higher expression in Cancer and vice versa. For statistical inference, each miRNA’s ΔCt values were compared between groups using Welch’s two-sample *t*-test to accommodate unequal variances and unpaired sampling. Raw *p*-values were corrected across assays by the Benjamini–Hochberg procedure, yielding false discovery rate (FDR)-adjusted *p*-values. Unless otherwise stated, miRNAs were retained for visualization and downstream target/pathway analysis if they satisfied both ∣log_2_(FoldReg)∣ > 1 and FDR < 0.05.

For connecting miRNAs to putative biological consequences, we assembled validated miRNA–mRNA interactions. For each direction (up- and down-regulated miRNAs), targets were retrieved with multiMiR::get_multimir [38] and restricted to the “validated” table, retaining only interactions supported as “Functional MTI” where available. Targets were mapped from HGNC symbols to Entrez Gene identifiers with clusterProfiler::bitr (OrgDb: org.Hs.eg.db) [39] and duplicate mappings were resolved to unique gene sets. To mitigate target-retrieval bias, the enrichment universe was the result of the union of validated targets across all retained miRNAs (i.e., the background against which each direction-specific set was tested). Gene Ontology Biological Process enrichment used clusterProfiler::enrichGO and the top 20 terms per direction were displayed as dot plots ordered by gene count. Finally, to summarize regulatory convergence on genes, we counted, within each direction, the number of distinct significant miRNAs that target each gene and plotted the top 20 genes as ranked bar charts. These counts were based on unique miRNA–gene pairs to avoid inflating support by repeated database entries. To control for potential confounding by clinical variables, we additionally performed multivariate linear modeling in R (lm) including age, BMI, and histological grade as covariates for the top dysregulated miRNAs.

To assess the sensitivity of our qPCR comparisons, we computed post hoc statistical power using the observed ΔCt dispersion. The minimal detectable effect size (MDES) was expressed on the log_2_(fold-change) scale for two-sample Welch *t*-tests, with per-sample normalization to the mean of three reference miRNAs (SNORD38B, U6 snRNA, SNORD49A). The representative dispersion was estimated as the median pooled SD of ΔCt across all non-housekeeping assays (SD ≈ 1.8). Familywise multiplicity was accounted for in two complementary ways: a conservative Bonferroni bound (α = 0.05/m, m = 84 assays) and a Benjamini–Hochberg (BH)-effective threshold α_eff = 0.05·(1 − π_0_), where π_0_ (the proportion of null hypotheses) was estimated from the observed *p*-value distribution. For each group contrast—Cancer vs. Healthy (n = 40 vs. 20) and POLE^+^ vs. POLE^−^ (n = 7 vs. 33)—we computed the MDES corresponding to 80% and 90% power under both α levels. These analyses were implemented in R using the pwr (version 1,3-0) and qvalue (version 2.38.0) packages, and summarized graphically as power versus effect-size curves.

In addition to the analyses on the qPCR data of cancer and healthy controls (n = 546 tumor and n = 33 control samples), publicly available miRNA sequencing data for uterine corpus endometrial carcinoma (TCGA-UCEC) were retrieved from the Genomic Data Commons (GDC) (accessed on 15 June 2025) using the TCGAbiolinks R package v2.234.0 [40]. Raw read counts for mature miRNAs were extracted, filtered to remove low-abundance features, and normalized using the edgeR package v4.4.2 [41]. A tumor versus normal differential expression analysis was performed with a negative binomial generalized linear model in edgeR, yielding log_2_ fold-change (log_2_FC), average expression, and false discovery rate (FDR) estimates for each miRNA. To compare the two datasets (qPCR and TCGA), overlapping miRNAs were identified by exact or stem-sequence matching. For each miRNA, the direction of regulation (up-regulated or down-regulated) was assessed in both datasets, and concordance was defined as agreement in the sign of the log_2_FC values.

## 5. Conclusions

Our study demonstrates that dysregulated miRNAs are not only biomarkers of EC but also key regulators of oncogenic signaling. While cancer overall is characterized by broad oncomiR up-regulation and loss of tumor-suppressive miRNAs, POLE-mutant tumors display a distinct profile marked by global down-regulation, reflecting alternative evolutionary strategies of tumor progression. These findings emphasize the biological and clinical relevance of miRNA shifts across EC subtypes. Future work integrating miRNA with transcriptomic and proteomic data, exploring circulating miRNAs as minimally invasive biomarkers, and testing functional roles in EC models will be critical. Ultimately, miRNA-based signatures may complement existing molecular classifiers, refining risk prediction and guiding personalized treatment strategies.

## Figures and Tables

**Table 1:** Demographic and clinicopathological characteristics of the study cohort.

	Endometrial Cancer (n = 40)	Control (n = 20)	*p* Values
**Age (years), mean ± SD**	63.25 ± 12.28	65.30 ± 9.26	0.43
**BMI (kg/m^2^), mean ± SD**	31.03 ± 8.59	27.37 ± 3.76	0.017
**Parity**	1.63 ± 1.06	2.20 ± 0.90	0.027
**Menopausal status**	36 (90%) postmenopausal	19 (95%) postmenopausal	0.61
**Histological type**			
Endometrioid	30 (75%)		
Serous	6 (15%)		
Clear cell	2 (5%)		
Mixed/Indetermined	2 (5%)		
**Tumour grade (FIGO)**			
G1 (Low)	18 (45%)		
G2 (Moderate)	9 (22.5%)		
G3 (High)	11 (27.5%)		
Mixed/Unspecified	2 (5%)		
**FIGO Stage**			
I	34 cases (85%)		
II	3 cases (7.5%)		
III	3 cases (7.5%)		
IV	0		
POLE mutation status	7 (17.5%) mutated	0	

**Table 2:** Primer sequences and product length.

Hotspot Mutations	Primer	Primer Sequence (5′-3′)	Product Length (bp)	Annealing Temperature (°C)
P286R (c.857C > G)	Forward (C)	GACCAAACTGCCCCTCAAGTTTCC	348	62
	Forward (G)	GACCAAACTGCCCCTCAAGTTTCG		
	Reverse	TGGCTGTCCTTCTGGAAGCCTAT		
V411L (c.1231G > C)	Forward (G)	CACATGGACTGCCTCAGGTGGG	188	63
	Forward (C)	CACATGGACTGCCTCAGGTGGC		
	Reverse	GTGGCGACAGCATCTGACACA		

## Data Availability

The data presented in this study are available on request from the corresponding author. The data are not publicly available due to privacy concerns.

## Supplementary Materials

The following supporting information can be downloaded at https://www.mdpi.com/article/10.3390/ijms262110438/s1.
